# Disability Worsening Phenotypes in Multiple Sclerosis and Impact of Disease-Modifying Treatments

**DOI:** 10.1212/WNL.0000000000214408

**Published:** 2025-11-24

**Authors:** Ermelinda De Meo, Ilaria Addazio, Emilio Portaccio, Raffaello Bonacchi, Matteo Betti, Francesco Patti, Simone Guerrieri, Matteo Foschi, Diana Ferraro, Pietro Annovazzi, Vincenzo Brescia Morra, Carla Tortorella, Alessandra Lugaresi, Federico Camilli, Carlo Pozzilli, Paola Perini, Franco Granella, Giovanna De Luca, Valentina Liliana Adriana Maria Torri Clerici, Marika Vianello, Silvia Romano, Eleonora E. Cocco, G. Lus, Alessia Di Sapio, Maria A. Rocca, Marta Simone, Pietro Iaffaldano, Massimo Filippi, Maria Trojano, Maria Pia Amato

**Affiliations:** 1 Queen Square Multiple Sclerosis Centre, Department of Neuroinflammation, UCL Queen Square Institute of Neurology, Faculty of Brain Sciences, University College London, United Kingdom;; 2 University of Studies of Florence-Department of Neurofarba, Firenze, Italy;; 3 Vita-Salute San Raffaele University, Milan, Italy;; 4 Dipartimento Scienze Mediche e Chirurgiche e Tecnologie Avanzate, GF Ingrassia, Università Catania UOS Sclerosi Multipla, Italy;; 5 Dipartimento di Neuroscienze–Centro Sclerosi Multipla–UO Neurologia–Ospedale S. Maria delle Croci–AUSL Romagna, Italy;; 6 Department of Neuroscience, Azienda Ospedaliero-Universitaria di Modena, Emilia-Romagna, Italy;; 7 Neurologia ad Indirizzo Neuroimmunologico–Centro Sclerosi Multipla–ASST della Valle Olona, Ospedale di Gallarate, Italy;; 8 Multiple Sclerosis Clinical Care and Research Center, Department of Neuroscience (NSRO), Federico II University, Naples, Italy;; 9 Centro Sclerosi Multipla–AO S.Camillo Forlanini, Roma, Italy;; 10 IRCCS Istituto delle Scienze Neurologiche di Bologna -Bologna, Italy;; 11 Dipartimento di Scienze Biomediche e Neuromotorie, Università di Bologna, Italy;; 12 Centro SM–Policlinico S. Andrea–Università Sapienza-Roma-Italy;; 13 Centro Specializzato Regionale per la Sclerosi Multipla; (CeSMuV), Regione Veneto, Dipartimento di Neuroscienze DNS, Azienda Ospedaliera, Università degli Studi di Padova, Italy;; 14 Centro Sclerosi Multipla–UOC di Neurologia–Dipartimento di Medicina Generale e Specialistica–AOU di Parma, Italy;; 15 Centro Sclerosi Multipla, Clinica Neurologica, Policlinico SS. Annunziata, Chieti, Italy;; 16 Neuroimmunology and Neuromuscular Diseases Unit, Fondazione IRCCS Istituto Neurologico Carlo Besta, Milan, Italy;; 17 Unit of Neurology, Cà Foncello Hospital, Treviso, Italy;; 18 Department of Neurosciences, Mental Health and Sensory Organs, Centre for Experimental Neurological Therapies (CENTERS), Sapienza University of Rome, Italy;; 19 Department of Medical Sciences and Public Health, Multiple Sclerosis Center, Binaghi Hospital, ASL Cagliari, University of Cagliari, Italy;; 20 Centro Clinico per la Sclerosi Multipla–II Clinica Neurologica–Università della Campania L. Vanvitelli, Napoli, Italy;; 21 Regional Referral MS Center and BioBank San Luigi Gonzaga University Hospital Orbassano (Torino), Italy;; 22 Neuropsychiatric Unit Department of Precision and Rigenerative Medicine and Jonic Area, University of Bari Aldo Moro, Italy;; 23 Centro SM -DiBraiN–Dipartimento di Biomedicina Traslazionale e Neuroscienze–Università di Bari, Italy; and; 24 IRCCS Fondazione Don Carlo Gnocchi, Florence, Italy.

## Abstract

**Background and Objectives:**

Patients with multiple sclerosis (MS) exhibit variability in disability progression and response to disease-modifying therapies (DMTs). Identifying those at greatest risk of disability worsening and most likely to benefit from high-efficacy DMTs remains challenging. We aimed to identify distinct disability worsening phenotypes, explore their mechanisms, and evaluate DMT impact across them.

**Methods:**

In this multicenter cohort study, we analyzed clinical and MRI data from propensity-matched cohorts of treated and untreated patients with relapse-onset MS from the Italian MS Register. Inclusion criteria were as follows: ≥3 years of follow-up, ≤1 year between disease onset and first assessment, and complete clinical and baseline MRI data. Latent class mixture models were applied to Expanded Disability Status Scale (EDSS) scores from untreated patients to identify disability worsening phenotypes. We compared proportions of progression independent of relapse activity (PIRA) and relapse-associated worsening events across phenotypes. A random forest algorithm, trained (70%) and tested (30%) on baseline clinical and MRI features of untreated patients, was used to assign phenotypes to treated patients. Linear mixed-effects models estimated DMT impact on disability trajectories within each phenotype.

**Results:**

We analyzed data from 2,563 untreated (mean age 41.2 ± 10 years, 67% female) and 2,952 treated (mean age 40.8 ± 11.4 years, 66% female) patients with MS over a median follow-up of 10.1 (interquartile range: 7.0–13.0) years. Four phenotypes were identified in untreated patients: “minimal-worsening” (15%), “late-worsening” (70%), “early-worsening” (3%), and “rapid-worsening” (12%). In all phenotypes, PIRA represented the main disability accrual mechanism. “Early-worsening” and “rapid-worsening” phenotypes exhibited more brain and spinal cord T2-hyperintense and gadolinium-enhancing lesions at baseline. The classification algorithm assigned phenotypes to patients receiving DMTs with 71% accuracy: “minimal-worsening” (18%), “late-worsening” (61%), “early-worsening” (13%), and “rapid-worsening” (8%). DMT exposure significantly reduced disability accrual in all phenotypes, with high-efficacy DMTs (β = −0.16, standard error (SE) = 0.06, *p* < 0.001) and early escalation (β = −0.18, SE = 0.06, *p* < 0.001) proving especially beneficial for the “rapid-worsening” phenotype.

**Discussion:**

We identified 4 clinically relevant disability worsening phenotypes in relapse-onset MS, primarily driven by PIRA, with greater CNS involvement linked to early and rapid progression. Despite reliance on EDSS alone, these phenotypes may inform personalized treatment and response assessment.

## Introduction

Multiple sclerosis (MS) is a chronic inflammatory demyelinating disease and a leading cause of neurologic disability among young adults.^1^ Traditionally, relapses are associated with disability accrual (relapse-associated worsening [RAW]). However, progression independent of relapse activity (PIRA) has gained increasing recognition over the past decade as an important contributor to long-term disability.^2,3^ This progression occurring early even in patients receiving disease-modifying treatments (disease-modifying therapies [DMTs]) suggests the existence of an ongoing, neurodegenerative component resistant to current interventions.^4^

On the contrary, variations in early disability accrual have been observed in patients with relapse-onset MS. Individual characteristics—including age, male sex, and extent of brain and spinal cord (SC) involvement—have been pinpointed as predictors of both recovery from relapses^4,5^ and long-term disability,^6^ suggesting the existence of multiple “disability worsening phenotypes.” Considerable efforts have been made to explain the biological basis of these variations. MRI-based subtypes^7^ and cognitive phenotypes^8^ with distinct biological underpinnings demonstrated that brain damage patterns correlate with clinical phenotypes beyond traditional descriptors. However, these classifications—derived from population‐level clustering—cannot be applied to individual patients and merely offer a cross‐sectional characterization of patient subgroups. Longitudinal progression has been inferred indirectly, either from subgroup prevalence across disease duration^8^ or through Subtype and Stage Inference (SuStaIn).^9^

The aims of this study were as follows: (1) to identify homogeneous groups of patients with relapse-onset MS who exhibited similar rates of disability worsening over time, as measured using the Expanded Disability Status Scale (EDSS), referred to as “disability worsening phenotypes”; (2) to assess the main drivers of disability progression within each phenotype; and (3) to evaluate the effects of DMTs on these phenotypes.

To achieve this, we applied latent class mixture modeling to longitudinal clinical data from a large cohort of untreated patients with relapse-onset MS from the Italian MS Register. We examined and compared the primary mechanisms underlying clinical disability accrual (RAW and PIRA) across these newly defined phenotypes, thereby providing insights into how relapse-associated and relapse-independent worsening differentially contribute to disability progression patterns within each subgroup. A machine learning algorithm was then trained and tested on the untreated cohort to identify predictors for each phenotype and to classify patients into disability worsening phenotypes based on baseline clinical and MRI features. This model was subsequently applied to an independent propensity-matched cohort of patients with MS receiving DMTs.

This approach enabled us to demonstrate a practical use-case of this complex modeling framework by assessing the impact of DMTs on disability accrual within each phenotype, comparing observed disability trajectories in treated patients with the trajectories expected based on the untreated cohort. Finally, we derived a “personalized treatment response index” to monitor treatment effect at the individual patient level.

## Methods

### Standard Protocol Approvals and Consents

The Italian MS Register was approved by local ethics committees, and written informed consent was obtained from all participants.

### Patients

Registered cases of adult (>18 years) relapse-onset MS were included, with disease onset between 1 January 1961 and 30 April 2021, and a minimum follow-up of 3 years before the study end date of 15 May 2024. Cases were excluded if there was a delay of more than 1 year between disease onset and the first clinical assessment, fewer than 3 EDSS measurements, missing key data (e.g., demographics, MS diagnosis/onset date, or baseline clinical and MRI features), significant record errors (e.g., inconsistent dates), or no EDSS scores recorded within the defined time window of 30 days before and 90 days after a documented clinical relapse. Analyses were conducted on patients with complete records.

### Data Collection

Baseline clinical information included sex, date of MS onset (first recorded clinical manifestation) and symptoms manifested, date of diagnosis, clinical course, and geographic region of residence with diagnosis based on the prevailing diagnostic criteria.^10-12^ Symptoms were grouped into 4 main localization categories: (1) optic nerve, (2) brainstem, (3) SC, and (4) hemispheric (including motor weakness or sensory disturbances not attributable to SC involvement, higher cortical dysfunctions, and visual field deficits not attributable to optic nerve involvement). Detailed information on disease phenotype, EDSS score (including functional system scores), relapses (date and degree of remission [complete/incomplete clinical recovery within 6 months or no remission]), and DMT use (product name, starting and stopping dates, and reasons for stopping) was recorded by the treating neurologist every 3 or 6 months and on occasions of clinical relapse evaluations. EDSS scores recorded within 30 days of a clinical relapse were excluded to avoid artificially inflating disability worsening. DMTs were categorized as moderate-efficacy (interferon-beta, glatiramer acetate, dimethyl fumarate, teriflunomide) or high-efficacy (natalizumab, cladribine, fingolimod, ozanimod, ponesimod, siponimod, rituximab, ocrelizumab, ofatumumab, alemtuzumab). To ensure consistent EDSS scoring, clinicians were required to complete mandatory certification, and data were centrally monitored for quality and accuracy.

MRI features collected in the Italian MS Register included scan date, CNS region scanned (brain/SC), number of T2-hyperintense lesions (categorized as 1–2, 3–8, or ≥9 in the brain and 0, 1–2, or ≥3 in the SC), the presence of T1-hypointense lesions (black holes), and the presence of gadolinium-enhancing (Gd+) lesions.

Based on EDSS, confirmed disability worsening (CDW) events were estimated, defined by an increase in EDSS score (either ≥1.5 points for patients with a baseline EDSS score of zero, ≥1.0 point for patients with a baseline EDSS score of 1.0–5.0, and 0.5 points for patients with a baseline EDSS score of ≥5.5). A 6-month confirmation interval was required for a CDW event to be considered valid.^13^ Only EDSS increases that were confirmed at a subsequent visit after this interval were included in the analysis. Then, CDW events were categorized as either RAW or PIRA. RAW was defined as a CDW event where the increase in disability occurred within 90 days after, or 30 days before, the onset of a relapse. PIRA was defined as a CDW event persisting for 6 months or longer, occurring more than 90 days after and more than 30 days before the onset of a relapse.^4^

### Statistical Analysis

#### Propensity Matching

As mentioned earlier, patients who had never received DMTs were analyzed separately to represent the untreated cohort of relapse-onset MS. Instead, patients receiving DMTs for a minimum of 3 months were propensity score–matched to the untreated cohort to minimize treatment allocation bias, based on baseline clinical, demographic, and MRI data. Matching was performed using the nearest-neighbor algorithm with a caliper width of 0.2 standard deviations of the logit of the propensity score, without replacement. A variable matching ratio was permitted to optimize covariate balance, resulting in unequal sample sizes between the treated and untreated groups. We weighted all subsequent analyses to account for the variable matching ratio, with the maximum cumulative weight for each matched patient being 1. After propensity score matching, patients receiving DMTs were classified as receiving moderate-efficacy or high-efficacy DMTs based on their first DMT. Patients who escalated DMT (switching from moderate to high-efficacy DMTs) were classified as escalating DMT.

#### Latent Class Mixture Models

Latent class mixture models identified distinct disability progression trajectories in untreated patients with MS. Ten models were fitted iteratively with an increasing number of latent classes (1–10). Optimal class number was determined using Akaike information criterion (AIC), Bayesian information criterion (BIC), sample-adjusted BIC (where lower values indicate better model fit), and the integrated log-likelihood (ICL) (where higher values indicate better model fit).^14,15^ To validate this model, a 10 k-fold cross-validation was performed. Clinical interpretability and parsimony were also considered.

#### Comparisons of Clinical and MRI Features Among Disability Worsening Phenotypes

To identify the primary mechanisms underlying disability worsening in the newly defined phenotypes, linear mixed-effects models were used to test the temporal relationship between disability accrual and annualized relapse rate (ARR). Subsequently, the χ^2^ test was applied to compare the contributions of RAW and PIRA to each disability worsening phenotype.

To further characterize the baseline features of the identified disability worsening phenotypes, demographic, clinical, and MRI variables at baseline were compared across groups. Chi-square and Fisher exact tests were used for categorical variables while the Student *t* test and Wilcoxon test were applied to continuous variables, as appropriate. Normality was assessed through visual inspection and the Kolmogorov-Smirnov test.

#### Random Forest Classification Algorithm

A random forest classification model was implemented to predict disability worsening phenotypes based on early clinical and MRI features. The cohort of untreated patients was randomly split into a training set (70%) and a test set (30%), and patients were classified into disability worsening phenotypes using their baseline clinical and MRI features. Variable importance in the random forest was assessed using permutation-based importance, expressed as the percentage decrease in model accuracy when each predictor was permuted. The trained model was then applied to patients with MS receiving DMTs to assign phenotypes.

#### Comparison of Expected vs Observed Disability Trajectories

For each patient receiving DMTs, predicted EDSS scores were calculated at each visit based on their assigned phenotype trajectory. Linear mixed-effects models assessed DMT impact—categorized as moderate efficacy, high efficacy, and escalating (for patients transitioning from moderate to high efficacy)—by comparing observed vs predicted EDSS score within each phenotype. We similarly evaluated how time to treatment initiation and time to DMT escalation influenced changes in disability severity. Finally, a “personalized treatment response index” was developed, using disability z-scores, calculated at each clinical assessment by comparing the patient's EDSS score with the mean and confidence intervals of their expected trajectory. Statistical significance was adjusted for multiple comparisons using the Bonferroni method, with a corrected *p* value threshold of <0.05. All analyses were performed using R software (version 4.3.2).

### Data Availability

The data sets used and analyzed during this study are available from the corresponding author on reasonable request.

## Results

### Demographic and Clinical Features

From 70,799 patients with MS included in the Italian MS Register, those meeting the inclusion and exclusion criteria were selected. After propensity score matching, treated patients were matched to the untreated cohort, resulting in 2 cohorts of 2,563 untreated patients and 2,952 patients receiving DMTs, balanced for baseline clinical, demographic, and MRI characteristics (Table 1).

**Table 1:** Demographic, Clinical, and Radiologic Features of Untreated Patients With MS and Those Receiving DMTs

	Untreated patients with MS	Patients with MS receiving DMTs	Untreated patients with MS vs patients with MS receiving DMTs, *p* values
N	2,563	2,952	—
Mean age at onset (SD) (y)	41.2 (10.1)	40.8 (11.4)	0.68^a^
Female/male	1729/834	1941/1,011	0.19^b^
Median EDSS score (IQR)	2.0 (1.0–2.5)	2.0 (1.0–2.5)	0.56^c^
Brainstem onset (%)	653 (25)	780 (26)	0.42^b^
Spinal cord onset (%)	761 (30)	919 (31)	0.28^b^
Optic nerve onset (%)	659 (26)	725 (25)	0.34^b^
Hemispheric onset (%)	802 (31)	885 (30)	0.29^b^
Presence of oligoclonal bands (%)	2,258 (88)	2,587 (88)	0.60^b^
Brain T2 lesion number (%)			0.09^b^
≥9	728 (29)	903 (31)	
3–8	1,393 (54)	1,519 (51)	
1–2	442 (17)	530 (18)	
Presence of brain Gd+ lesions (%)	1,232 (48)	1,407 (48)	0.76^b^
Spinal cord T2 lesion number (%)			
≥3	193 (8)	229 (7)	0.07^b^
1-2	1,387 (54)	1,679	
0	983 (38)	1,044	
Presence of spinal cord Gd+ lesions (%)	947 (37)	1,041 (35)	0.19^b^
Median follow-up duration (IQR)	10.1 (7.3–13.7)	8.9 (6.5–12.8)	<0.001

Abbreviations: DMTs = disease-modifying therapies; EDSS = Expanded Disability Status Scale; Gd+ = gadolinium-enhancing; IQR = interquartile range; MS = multiple sclerosis.

a Student *t* test.

b χ² test.

c Kruskal-Wallis test.

#### Disability Worsening Phenotypes

Using latent class mixture modeling, a 4-class model was identified as the best fit for describing disability worsening in the untreated cohort.

The AIC, BIC, sample-adjusted BIC, and ICL values for models ranging from 1 to 10 classes are presented in Table 2. In addition, we obtained cross-validation results, which are provided in eTable 1.

**Table 2:** Model Summary for Classes From 1 to 6

Number of classes	AIC	BIC	SABIC	ICL
1	91,221.11	91,290.83	91,252.70	91,290.83
2	89,378.25	89,471.21	89,420.38	90,058.42
3	88,337.34	88,453.54	88,389.99	89,361.65
4	88,015.38	88,154.82	88,078.56	89,536.29
5	89,765.97	89,928.65	89,839.68	89,838.14
6	89,865.97	90,128.65	89,939.68	90,038.14

Abbreviations: AIC = Akaike information criterion; BIC = Bayesian information criterion; ICL = integrated log-likelihood; SABIC = sample-adjusted Bayesian information criterion.

**Figure 1:**
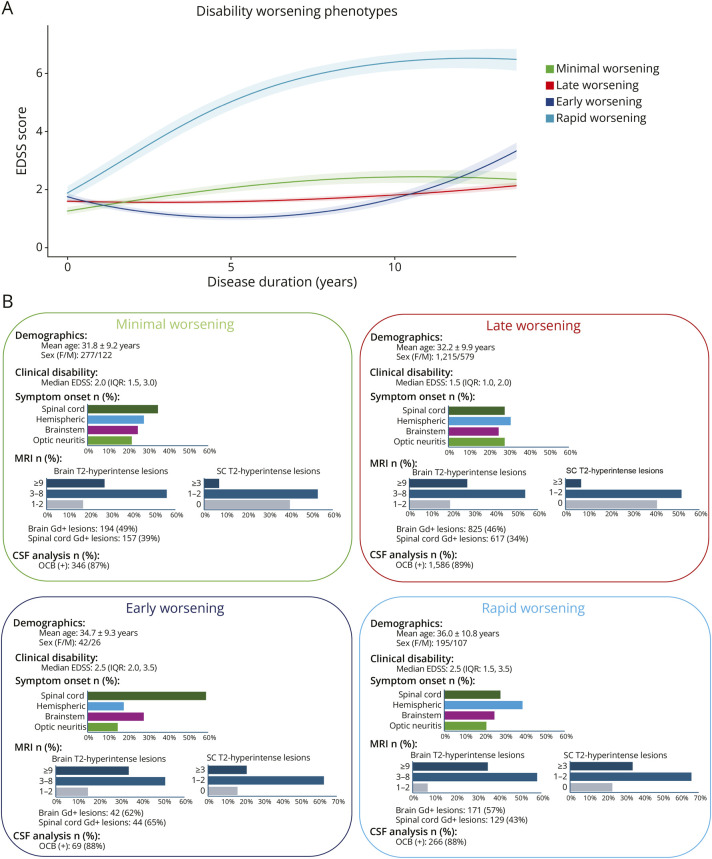
Disability Worsening Phenotypes In panel A, EDSS trajectories over 15 years of disease duration are plotted for the 4 disability worsening phenotypes. Color codes: dark blue, “minimal-worsening”; red, “late-worsening”; green, “early-worsening”; and light blue, “rapid-worsening.” Solid lines represent the mean trajectories with shaded confidence intervals. Panel B summarizes the demographic, clinical, MRI, and CSF characteristics of patients exhibiting disability worsening phenotypes at disease onset. Data are stratified by the 4 disability worsening phenotypes. For each phenotype, the following are displayed: demographics: mean age (± SD) and sex distribution (female/male); clinical presentation: median (Expanded Disability Status Scale [EDSS; interquartile range [IQR]) score at onset and proportion of patients presenting with optic neuritis, transverse myelitis, or other CNS syndromes; MRI characteristics: percentage of patients with brain T2 brain (1–2, 3–8, ≥9) lesions and spinal cord (0, 1–2, ≥3) lesions, gadolinium-enhancing (Gd^+^) lesions in the brain or spinal cord); CSF analysis: percentage of patients with oligoclonal bands (OCBs). EDSS = Expanded Disability Status Scale; Gd+ = gadolinium-enhancing; IQR = interquartile range; OCBs = oligoclonal bands; SC = spinal cord.

The 4 identified groups—hereafter referred to as “disability worsening phenotypes”—were named based on the timing of disability worsening onset as derived from their modeled trajectories (Figure 1, Panel A):

1. Minimal-worsening (n = 399, 15%): characterized by very slow and mild disability progression, reaching a plateau at an EDSS score of 2 around 10 years after disease onset.
2. Late-worsening (n = 1,794, 70%): disability progression beginning approximately 10 years after disease onset.
3. Early-worsening (n = 68, 3%): disability progression starting around 5 years after disease onset.
4. Rapid-worsening (n = 302, 12%): marked by severe and rapid disability progression from the time of disease onset.

### Baseline Characteristics of Disability Worsening Phenotypes

We compared clinical and demographic features at disease onset among disability worsening phenotypes (Figure 1 Panel B, eFigure 1 and eTable 2). Patients with “minimal-worsening” and “late-worsening” phenotypes had similar age (mean [SD] age, 31.8 [9.2] years and 32.2 [9.9] years) and EDSS scores (median interquartile range [IQR] 2.0 [1.5, 3.0] and 1.5 [1.0, 2.0]) at disease onset. They were younger and had lower EDSS scores at disease onset compared with patients belonging to “early-worsening” (mean [SD] age, 34.7 [9.3] years, *p* = 0.02; median [IQR] EDSS score, 2.5 [2.0, 3.5]; *p* < 0.001) and “rapid-worsening” (mean [SD] age, 36.0 [10.8] years; median [IQR] EDSS score, 2.5 [1.5, 3.5]; *p* < 0.001) phenotypes.

Compared with all remaining phenotypes, patients with the “late-worsening” phenotype more frequently experienced optic neuritis at disease onset (28% vs 22% in minimal-worsening, *p* = 0.03; 15% in early-worsening, *p* = 0.02; 21% in rapid-worsening, *p* = 0.01). Patients with the “early-worsening” phenotype more frequently experienced onset with SC symptoms (59% vs 35% in “minimal-worsening,” *p* = 0.004; 28% in “late-worsening,” *p* < 0.001; 28% in “rapid-worsening,” *p* < 0.001). Patients with the “rapid-worsening” phenotype more frequently experienced hemispheric onset (39% vs 28% in “minimal-worsening,” *p* = 0.003; 31% in “late-worsening,” *p* = 0.03; 18% in “early-worsening,” *p* < 0.001).

Regarding MRI features, patients with the “early-worsening” phenotype more frequently had Gd+ brain lesions (62%) than those with “minimal-worsening” (48%, *p* = 0.05) and “late-worsening” (46%, *p* = 0.05) phenotypes. They more frequently had Gd+ SC lesions (65%) compared with those with other phenotypes (vs 39% in “minimal-worsening,” *p *= <0.001; 34% in “late-worsening,” *p* < 0.001; and 43% in “rapid-worsening,” *p* < 0.001), and a higher proportion of patients had at least 1 SC lesion (84% vs 60% in “minimal-worsening,” *p *= <0.001; 59% in “late-worsening,” *p* < 0.001; 77% in “rapid-worsening,” *p* = 0.04).

Compared with those with “minimal-worsening” and “late-worsening” phenotypes, a higher proportion of patients with “rapid-worsening” phenotype had 9 or more brain T2-hyperintense lesions (35% vs 27% in “minimal-worsening,” *p* < 0.001; 27% in “late-worsening,” *p* < 0.001), at least 1 brain Gd+ lesion (57% vs 49% in “minimal-worsening,” *p* = 0.04; 46% in “late-worsening,” *p* < 0.001), and at least 1 SC lesion (77% vs 60% in “minimal-worsening,” *p *= <0.001; 59% in “late-worsening,” *p* < 0.001).

**Figure 2:**
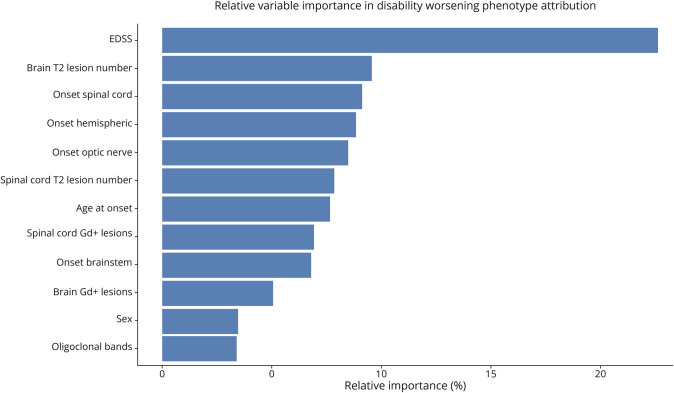
Relative Variable Importance for Phenotype Attribution Relative variable importance in attributing disease worsening phenotypes. EDSS = Expanded Disability Status Scale; Gd+ = gadolinium-enhancing.

The random forest classification algorithm was applied on baseline clinical and MRI features aiming to identify which disability worsening phenotype a patient would belong to at the onset of the disease with an accuracy of 71%. Among the most relevant variables, baseline EDSS score, number of T2-hyperintense lesions (brain and SC), and symptom onset location (SC, hemispheric, optic nerve, or brainstem) accounted for 70% of the classification into the 4 disability phenotypes. We defined variable importance in the whole group (Figure 2) and in predicting individual classes (eFigure 2).

### Disability Worsening Phenotypes: ARR and CDW Events

Patients with the “rapid-worsening” phenotype had higher mean ARR compared with the remaining phenotypes (0.36 ± 0.31 vs 0.23 ± 0.19, *p* < 0.001 in “minimal-worsening,” vs 0.24 ± 0.20, *p* < 0.001 in “late-worsening,” and vs 0.28 ± 0.19, *p* = 0.01 in “early-worsening”). To explore whether relapse activity influenced disability progression, the relationship between ARR and EDSS score increases was examined. An association between the rate of EDSS score increase and ARR reduction over time was observed in the “minimal-worsening” (ARR × time interaction: β = −0.25, standard error (SE) = 0.10, *p* = 0.02) and in “early-worsening” (ARR × time interaction: β = −0.24, SE = 0.03, *p* < 0.001) phenotypes. No associations between ARR and EDSS worsening rate were observed in the remaining phenotypes.

**Figure 3:**
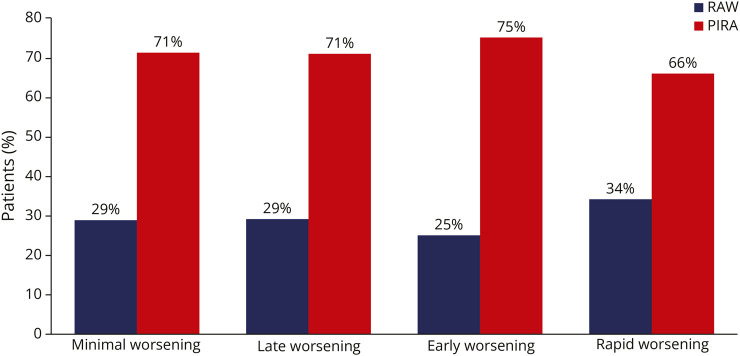
Contribution of RAW and PIRA to Disability Accrual in Disability Worsening Phenotypes Histograms report the percentage of relapse-associated worsening (RAW) (blue) and of progression independent of relapse activity (PIRA) (red) events among the newly defined phenotypes.

Furthermore, CDW events were examined and classified as RAW or PIRA. In the untreated cohort, 43% of patients experienced at least 1 CDW event, with a total of 1,846 events recorded. The risk of an all-cause CDW event was highest in the “rapid-worsening” phenotype (“minimal-worsening” = 42%, “late-worsening” = 36%, “early-worsening” = 37%, “rapid-worsening” = 84%). PIRA was the primary driver of disability worsening across all newly defined phenotypes, with no statistically significant differences between them (Figure 3).

### Disability Worsening Phenotypes in Patients Receiving DMTs

By applying the model trained and tested on the untreated cohort, patients with MS receiving DMTs were classified as follows: “minimal-worsening” (n = 531, 18%), “late-worsening” (n = 1,800, 61%), “early-worsening” (n = 383, 13%), and “rapid-worsening” (n = 238, 8%).

### DMT Effect on Disability Worsening Phenotypes

Predicted and observed disability (EDSS) trajectories in patients receiving DMTs were grouped by DMT strategy (moderate-efficacy, high-efficacy, escalation) and compared (Table 3 and eFigure 3). In the minimal-worsening phenotype, all DMT strategies reduced disability accrual over time, with diminishing effects later for moderate-efficacy and escalation strategies. Earlier treatment significantly improved outcomes only with moderate-efficacy DMTs.

**Table 3:** Comparisons Between Predicted and Observed Disability Worsening Trajectories in Patients With Relapse-Onset MS Grouped According to Disability Worsening Phenotype and DMTs

	Moderate-efficacy DMTs		Escalation DMTs		High-efficacy DMTs	
	β coef (SE)	*p* Values	β coef (SE)	*p* Values	β coef (SE)	*p* Values
Minimal worsening	N = 295		N = 16		N = 216	
Observed vs predicted × time	−0.11 (0.04)	0.01	−0.15 (0.04)	<0.001	−0.41 (0.20)	0.05
Observed vs predicted × time^2^	0.35 (0.03)	<0.001	0.11 (0.04)	<0.001	0.12 (0.17)	0.51
Observed vs predicted × time to treatment/^a^time to switch effect × time	−0.31 (0.11)	0.02	−0.14 (0.10)	0.20	1.01 (0.56)	0.11
Observed vs predicted × time to treatment/^a^time to switch effect × time^2^	0.17 (0.08)	0.06	0.27 (0.08)	<0.001	−0.52 (0.46)	0.33
Late worsening	N = 1,085		N = 225		N = 474	
Observed vs predicted × time	0.24 (0.15)	0.14	0.41 (0.03)	<0.001	0.16 (0.05)	<0.001
Observed vs predicted × time^2^	−0.51 (0.02)	<0.001	−0.38 (0.02)	<0.001	−0.26 (0.04)	<0.001
Observed vs predicted × time to treatment/^a^time to switch effect × time	−0.07 (0.05)	0.27	0.15 (0.06)	0.01	0.35 (0.24)	0.19
Observed vs predicted × time to treatment/^a^time to switch effect × time^2^	−0.03 (0.04)	0.49	−0.16 (0.04)	<0.001	−0.29 (0.16)	0.10
Early worsening	N = 193		N = 43		N = 138	
Observed vs predicted × time	0.61 (0.05)	<0.001	0.50 (0.04)	<0.001	0.13 (0.12)	0.27
Observed vs predicted × time^2^	−0.63 (0.04)	<0.001	−0.46 (0.04)	<0.001	−0.30 (0.10)	<0.001
Observed vs predicted × time to treatment/^a^time to switch effect × time	−0.46 (0.14)	<0.001	−0.06 (0.09)	0.65	−1.05 (0.27)	<0.001
Observed vs predicted × time to treatment/^a^time to switch effect × time^2^	0.32 (0.10)	<0.001	−0.30 (0.07)	<0.001	1.23 (0.19)	<0.001
Rapid worsening	N = 27		N = 102		N = 110	
Observed vs predicted × time	−0.24 (0.15)	0.14	−0.18 (0.06)	<0.001	−0.16 (0.06)	<0.001
Observed vs predicted × time^2^	0.21 (0.12)	0.09	0.08 (0.04)	0.09	−0.09 (0.06)	0.16
Observed vs predicted × time to treatment/^a^time to switch effect × time	−1.23 (0.50)	0.02	−0.12 (0.13)	0.42	−1.35 (0.23)	<0.001
Observed vs predicted × time to treatment/^a^time to switch effect × time^2^	0.68 (0.32)	0.04	0.29 (0.11)	0.01	0.83 (0.15)	<0.001

Abbreviations: coef = coefficient; DMTs = disease-modifying therapies; SE = standard error.

We excluded 28 patients from this analysis because they were receiving off-label treatments not approved for MS (azathioprine, n = 4; cyclophosphamide, n = 8; mitoxantrone, n = 12, IV immunoglobulin, n = 4).

The effect of time to treatment and time to DMT switch was also tested.

a For patients undergoing DMT escalation.

In the late-worsening phenotype, disability initially accrued faster than expected under escalation and high-efficacy DMTs but significantly slowed over time across all DMT strategies. Earlier escalation positively influenced disability trajectories while time to treatment showed no effect for moderate-efficacy or high-efficacy DMTs.

In the early-worsening phenotype, moderate-efficacy and escalation strategies showed initially faster worsening, but all strategies slowed disability accumulation later. Earlier treatment initiation significantly reduced disability accrual for moderate-efficacy and high-efficacy DMTs.

In the rapid-worsening phenotype, escalation and high-efficacy DMTs significantly slowed disability accumulation while moderate-efficacy DMTs did not. Earlier treatment initiation improved outcomes for moderate-efficacy and high-efficacy DMTs, although these benefits gradually diminished.

**Figure 4:**
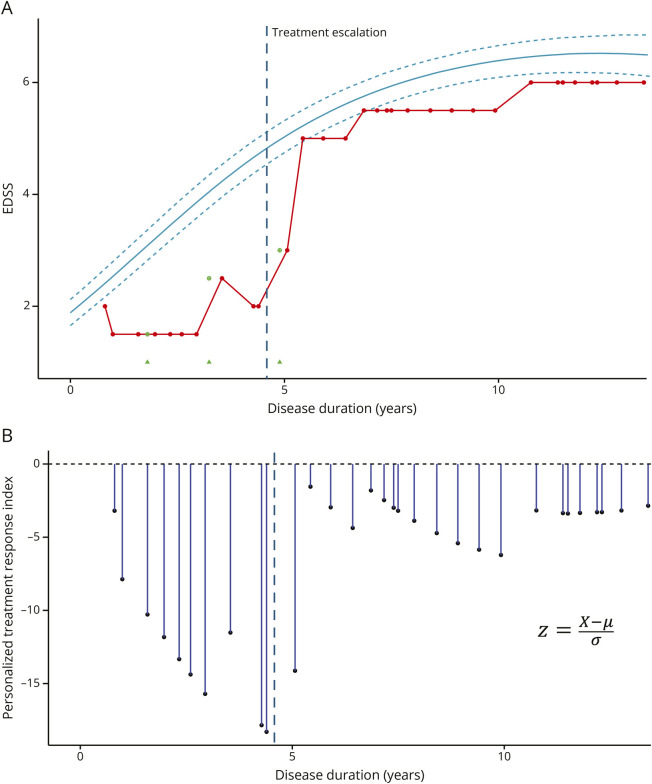
Example of an Individual Patient Trajectory In panel A, the solid line represents the mean predicted Expanded Disability Status Scale (EDSS) trajectory while dashed lines represent its 95% CI. Each EDSS score of the patient collected after a minimum of 30 days from relapses is represented as a red dot: EDSS scores during relapses are represented as green circles with a cross inside. Relapses are indicated as green triangles. Panel B reports the personalized treatment response index over the disease duration.

Finally, an example of the “personalized treatment response index” was provided, the “disability z-score,” showing the predicted vs observed EDSS trajectories at individual patient level (Figure 4).

## Discussion

This study used latent class mixture models to identify distinct disability progression trajectories in patients with relapse-onset MS. Four phenotypes were delineated: “minimal-worsening,” “late-worsening,” “early-worsening,” and “rapid-worsening.” Baseline clinical and MRI features allowed phenotype assignment through machine learning with 71% accuracy. To demonstrate the model's clinical applicability, we assessed the effect of DMTs on disability worsening trajectories and calculated a patient-specific “disability z-score” quantifying deviation of observed disability from predicted trajectory.

The “minimal-worsening” phenotype exhibited low and stable disability resembling previously defined “benign MS.”^16,17^ These patients had fewer brain and SC lesions^18-21^ and a younger age at onset, reflecting greater resilience.^22^ We chose the term “minimal-worsening” over “benign MS” because up to 30% of such patients develop cognitive impairment or worsening in other domains not captured by EDSS (e.g., fatigue, mood, and upper limb dysfunction),^23^ which cannot be considered “benign.” Instead, “minimal-worsening”, albeit imperfect, better reflects the concept of EDSS stability, not considering changes in cognition or other functions. Moreover, we observed that DMTs reduced disability accrual over time in this phenotype, suggesting ongoing inflammatory activity and inflammation-related neurodegeneration,^24^ which reinforces the rationale for initiating DMTs even in patients with low levels of disability.^25^ Of interest, this beneficial effect declined over time, supporting potential DMT discontinuation strategies later in the disease course. Recent evidence from de-escalation and discontinuation trials indicates that DMT discontinuation may be safe in carefully selected patients aged ≥55–60 years with ≥5 years of stable disease without relapses or MRI activity, particularly on platform therapies.^26^ Thus, it is tempting to speculate that early introduction of DMTs may be sufficient to partially interrupt the cascade of inflammation-associated neurodegeneration in this specific subgroup of patients.

The “late-worsening” phenotype showed low disability levels for the first 10 years, followed by subsequent accrual. It was the most common phenotype in both untreated (70%) and treated (61%) cohorts, indicating that most clinical trials and research studies include these patients. This may limit the generalizability of trial results, underscoring the need for stratified analyses in future studies that also account for individuals with earlier or more severe progression patterns. At disease onset, the “late-worsening” phenotype showed a higher frequency of optic neuritis (∼30% in line with natural history studies),^27,28^ were younger, and had a lower lesion load compared with the more severe phenotypes. The known favorable prognostic impact of MS onset with optic neuritis^27,28^ might reflect a shorter subclinical phase, as evidenced by the lower CNS lesion load at presentation. This aligns with other studies^21^ showing that long-term disability and conversion to secondary progression are driven more by brain and SC MRI abnormalities than by symptom localization.^21^ In this phenotype, patients on DMTs showed initially faster disability worsening, followed by stabilization or slowing over time. This may reflect unmeasured negative prognostic factors not accounted for in our propensity matching; however, DMTs ultimately reduced disability accrual.

The “early-worsening” phenotype showed low disability scores during the first 5 years, followed by rapid worsening, accounting for 3% of the untreated and 13% of treated patients. It was characterized by extensive SC involvement, with 74% of patients exhibiting at least 1 lesion and 65% Gd+ lesions. Spinal cord lesions at disease onset are known to predict conversion to MS from both radiologically^29,30^ and clinically isolated syndromes,^31,32^ as well as long-term disability.^21^ Possible explanations are the limited reparative capacity of this structure^33^ and the concentration of pathways essential for locomotion, sensation, and sphincter control. Although we could not assess cord atrophy, it is plausible that atrophy resulting from retrograde and anterograde degeneration starting from lesions at distant sites may contribute to the disability progression observed in this phenotype, in line with the well-established association between SC atrophy and disability progression.^34,35^ This phenotype also showed a higher proportion of brain Gd+ lesions. Although their prognostic value for long-term disability remains debated,^21,36,37^ these findings align with studies linking early inflammatory activity to long-term disability and secondary progression.^38^ Of interest, in this phenotype, an inverse relationship between ARR and EDSS score increase over time was observed, suggesting that compensatory mechanisms may preserve function until CNS reserves are exhausted. Patients on DMTs initially worsened faster, especially with moderate-efficacy DMTs, but disability worsening slowed over time across all DMT strategies, highlighting their disease-modifying effect. Timely initiation and escalation further improved outcomes, underscoring the need to early identify patients with more severe disability phenotypes to optimize therapy.^25,39^

The “rapid-worsening” phenotype, including 12% of untreated patients with MS and 8% of patients with MS receiving DMTs, showed rapid EDSS score increase from disease onset. This phenotype was characterized by higher brain lesion load compared with all remaining phenotypes and SC lesion load compared with “minimal-worsening” and “late-worsening” phenotypes. Baseline number and volume of brain^21,40-42^ and SC lesions^21,43,44^ are established predictors of poor prognosis, reflecting early neuroaxonal damage. Furthermore, the frequent hemispheric onset in patients with “rapid worsening” suggests not only a higher lesion volume but also a predilection for lesions in critical areas of the CNS, such as motor and sensory pathways, contributing to faster disability progression. In this phenotype, high-efficacy DMTs and escalation strategies significantly slowed disability progression over time, whereas moderate-efficacy DMTs had no impact. Earlier treatment initiation further reduced disability accumulation, although this benefit waned over time, underscoring both the relevance of early and aggressive treatment strategies to prevent long-term disability^33^ and the need to develop neuroprotective therapies to address long-term neurodegenerative processes.

The definition of homogeneous subgroups of MS patients with divergent disease trajectories is a step forward in personalized medicine for MS.^45,46^ Among previous studies with this aim, Eshaghi et al.^47^ inferred long-term progression patterns based on cross-sectional MRI data (brain volume loss, lesion accumulation, and normal-appearing white matter disruption). Unfortunately, our results are not directly comparable because of the unavailability of MRI scans for our cohort. Key strengths of our study include its longitudinal design and the use of routinely collected EDSS data. However, reliance on the EDSS alone prevented us from comparing our disability trajectories with recently described trajectories in physical and mental health–related quality of life.^46^ Another notable advantage is that our trajectories were derived from untreated patients, thereby avoiding treatment-related confounding. However, the phenotype distribution in our DMT-treated cohort aligns with that of a previous group-based trajectory modeling study^45^ of 201 patients with relapse-onset MS on DMTs, which identified 3 worsening groups: approximately 60% with moderate disability (median EDSS score 2.5 over 10 years), similar to our “late-worsening” phenotype; and the rest split between minimal/no progression and severe disability, paralleling our “minimal-worsening” and combined “early-worsening” and “rapid-worsening” phenotypes. Our larger sample size and longer follow-up enabled a more granular identification of disability trajectories.

Furthermore, we investigated the mechanisms of disability accumulation by examining the contributions of RAW and PIRA within each phenotype. The “rapid-worsening” phenotype showed a higher number of CDW events and a trend toward a greater proportion of RAW events (34% vs 25%–29% in other phenotypes), supporting the role of severe inflammatory damage in this group. However, the difference did not reach statistical significance, possibly because the initial impact of relapses may be masked by transient reparative mechanisms, which later fail, resulting in PIRA driving long-term disability. Indeed, PIRA emerged as the dominant mechanism driving CDW across all phenotypes, suggesting that neurodegeneration is the primary contributor to permanent disability. It is important to note that while PIRA aims to capture progression unrelated to relapses, its operational definitions vary. There is ongoing debate on re-baselining EDSS after relapses, optimal confirmation periods to minimize score variability, and the temporal separation needed to define events as relapse-independent.^3,48^ Within this framework, it should be considered that the definition of PIRA used in this study—aligned with our previous work using the same cohort^2,49^—may have influenced the reported findings.

Finally, to further prove the applicability of our disability progression phenotypes in the clinical practice, a “disability z-score” was calculated reflecting the difference between the observed and expected disability and providing a measure at patient level of treatment strategy efficacy. On further validation, this z-score could be used in clinical trials, thus overcoming the potential biases due to hidden heterogeneities within the MS cohort.

This study has several limitations. First, our identified disability worsening phenotypes rely solely on EDSS scores, which do not capture subtle clinical changes, cognitive impairment, fatigue, mood alterations, or upper limb dysfunction, all of which can significantly influence disability without necessarily affecting the EDSS. Still, given the EDSS score remains the most widely used measure, our findings remain generalizable. Moreover, while we cannot exclude that our cohort of patients not receiving DMTs may be characterized by milder disability, there is evidence supporting that a large proportion of patients (∼80%) maintain an EDSS score of ≤2 over approximately 15 years of follow-up.^21,45,50^ Although the MS Registry lacks data on clinical reasons for treatment decisions, many untreated patients were diagnosed in the 1990s, when DMT availability was limited. We also lacked data on cardiovascular risk factors and smoking—known contributors to disability progression—and had no direct access to MRI scans, thus precluding assessment of scanner differences, lesion location, brain and SC atrophy, chronic active lesions, and other advanced MRI biomarkers. Similarly, neuropsychological evaluations were unavailable, limiting assessment of cognitive impairment. Our disability worsening phenotypes were defined in a single, albeit large and multicentric, cohort; therefore, replication in independent data sets and clinical trial settings is necessary. Another limitation is that these phenotypes were retrospectively derived from long-term disability trajectories, limiting their immediate clinical applicability. Nonetheless, our baseline feature–based classifier provided proof-of-principle for prospective identification, albeit with modest accuracy. This model requires external validation and integration with fluid or advanced imaging biomarkers to achieve sufficient precision for individual-level use. Owing to the stringent inclusion criteria for EDSS availability and follow-up assessments, the final study cohort may differ from the broader Italian MS population. Finally, the limited number of patients receiving each specific DMT—or even within broader therapeutic categories (e.g., anti-CD20 therapies vs S1P modulators)—precluded reliable between-group comparisons. Although our study was not powered to address treatment effects on PIRA across disability worsening phenotypes, this may represent an important avenue for future research.

In conclusion, we identified 4 distinct disability worsening phenotypes in relapse-onset MS: minimal-worsening (very slow progression reaching EDSS score 2 after approximately 10 years), late-worsening (progression begins around 10 years after onset), early-worsening (progression starting at approximately 5 years), and rapid-worsening (substantial immediate disability accumulation). Consistent with previous studies, the more severe phenotypes exhibited MRI evidence of widespread brain and SC involvement. PIRA emerged as the predominant mechanism driving progression across all phenotypes, underscoring the urgent need for therapies targeting chronic neuroinflammation and neurodegeneration. As proof of concept, we showed that these phenotypes can be assigned to individual patients, enabling objective comparison of observed vs expected disability over time by deriving “disability z-scores.” This advances classical phenotyping toward more accurate, patient-centered categories integrating routine MRI features. Clinically, these phenotypes may aid personalized treatment selection and monitoring, provided adequate validation; in research, they represent a potentially useful framework for studying disability progression and treatment response.

## Glossary

AIC: Akaike information criterion
ARR: annualized relapse rate
BIC: Bayesian information criterion
CDW: confirmed disability worsening
DMT: disease-modifying therapy
EDSS: Expanded Disability Status Scale
ICL: integrated log-likelihood
IQR: interquartile range
MS: multiple sclerosis
PIRA: progression independent of relapse activity
RAW: relapse-associated worsening
SC: spinal cord
SE: standard error
